# Opportunities for Participation in Randomized Controlled Trials for Patients with Multiple Myeloma: Trial Access Depends on Restrictive Eligibility Criteria and Patient Expectations

**DOI:** 10.3390/cancers14092147

**Published:** 2022-04-26

**Authors:** Amelie Boquoi, Veronika Rings, Annemarie Mohring, Ingrida Savickaite, Romans Zukovs, Judith Strapatsas, Kathrin Nachtkamp, Guido Kobbe, Ulrich Germing, Roland Fenk

**Affiliations:** Department of Hematology, Oncology and Clinical Immunology, University Hospital Duesseldorf, Heinrich Heine University, 40225 Duesseldorf, Germany; veronika.rings@hhu.de (V.R.); annemarie.mohring@med.uni-duesseldorf.de (A.M.); ingrida.savickaite@med.uni-duesseldorf.de (I.S.); romans.zukovs@med.uni-duesseldorf.de (R.Z.); judith.strapatsas@med.uni-duesseldorf.de (J.S.); kathrin.nachtkamp@med.uni-duesseldorf.de (K.N.); kobbe@med.uni-duesseldorf.de (G.K.); germing@med.uni-duesseldorf.de (U.G.); fenk@med.uni-duesseldorf.de (R.F.)

**Keywords:** multiple myeloma, randomized clinical trials, eligibility, real-world

## Abstract

**Simple Summary:**

Over the past decade, randomized controlled trials as an established instrument of evidence-based medicine have contributed fundamentally to the development and approval of new substances. However, it has been frequently shown that less than 5% of adult cancer patients enroll in clinical trials. Barriers to trial participation have been extensively studied, but the rate of trial participation has not changed substantially. In this retrospective analysis, we found that 53% of newly diagnosed multiple myeloma patients met the eligibility criteria, while only 38% of relapsed refractory patients were eligible. Moreover, our data show for the first time that eligible patients tend to become more reluctant to participate over the course of the disease, with 42% of newly diagnosed patients consenting, and only 7% of relapsed/refractory patients consenting. Thus, our results may assist with trial design improvements and address patient expectations and priorities in order to increase enrollment.

**Abstract:**

Randomized controlled trials (RCT) are the driver of therapeutic innovations. However, it has been frequently shown that less than 5% of adult cancer patients enroll in clinical trials, although 70% of patients are considered as being willing to participate. Barriers to trial participation have been extensively studied. Although there is evidence that trial participation correlates with improved survival and reduced mortality, the rate of participation has not changed substantially. We provide retrospective data from a single-center analysis of 411 patients with multiple myeloma (MM) who were treated at the University Hospital Duesseldorf in Germany between January 2014 and December 2016. Each patient was analyzed for the real-world possibility of participating in a clinical study, based on the inclusion and exclusion (I/E) criteria and the recruiting period of open studies. The overall rate of study participation was 19%. A total of 53% of NDMM patients were eligible for first-line studies (GMMG-HD6, LenaMain). Of these, 80% consented to enrolment (42% of all). In contrast, only 38% of the RRMM population was eligible (GMMG-Relapse, Castor, Tourmaline, Admyre). Of these, only 22% (7% of all) consented. This was confirmed by virtual analysis, showing that only 29% of all RRMM patients would have been eligible for six internationally recruiting trials leading to later drug approval. The majority of cases were rendered ineligible by only one I/E criterion. The most common criteria were study-specific (prior therapies or refractory disease to a specific drug), kidney disease, and previous malignancy, followed by internal, neurologic, and infectious disease. In summary, this single-center analysis showed that I/E criteria permit study participation for most NNDM patients, with a dramatic decrease in the RRMM population. This is aggravated by the fact that the willingness for study participation also significantly declines in RRMM. Thus, addressing patient expectations and priorities seems to be the most promising approach to increasing patient enrollment in clinical trials.

## 1. Introduction

Multiple myeloma (MM) is a clonal plasma cell neoplasia and accounts for 10% of malignant hematological tumors [1]. Over the past decade, randomized controlled trials (RCTs) as an established instrument of evidence-based medicine have contributed fundamentally to the development and approval of new substances, thus leading to considerable medical progress. Several large phase III clinical trials have been performed for both newly diagnosed and relapsed/refractory patients, in order to improve treatment efficacy, offer better convenience, or minimize side effects [2,3,4,5].

Eligibility criteria have been established to assess the efficacy in a defined population and protect the safety of the trial participants [6]. However, numerous authors have estimated that less than 5% of adult cancer patients enroll in clinical trials, although 70% of patients are considered as being willing to participate [7,8,9,10]. Several barriers to enrolment have been identified and can be broadly categorized as patient-specific (both disease-related or due to patient concern or lack of interest), protocol-specific (eligibility criteria, time demands, or availability), or physician-specific (physician bias) [11,12].

Experience with cytotoxic chemotherapeutics has shaped common inclusion and exclusion criteria over time, and these criteria are often duplicated from previous studies as a start or a template for the next trial. Instead, criteria should be modified individually to meet the objectives of each study, with consideration being made toward the anticipated safety of the investigational agent in the new study, or the ability to recruit trial participants from the patient population [13,14]. Therefore, evaluating the trial design and inclusion and exclusion criteria, as well as patient preferences, could help to identify opportunities to improve studies in such a way that increases patient enrolment and ensures that study participants are more representative of the general multiple myeloma population [15].

## 2. Materials and Methods

We retrospectively analyzed 634 patients with multiple myeloma who were treated at the University Hospital Düsseldorf in Germany from 31 January 2014 to 31 December 2016.

Each patient in that 3-year time frame was reviewed for the possibility of participating in a therapy study with two different methods (Figure S1).

Our first analysis was based on real-world data from a cohort of newly diagnosed multiple myeloma (NDMM) and relapsed/refractory multiple myeloma (RRMM) patients, which were extracted from clinical trials conducted at our department. This included the first-line trials LenaMain (EudraCT: 2007-003945-33) and GMMG-HD6 (EudraCT: 2014-003079-40). The second-line trials comprised Relapse (EudraCT: 2009-013856-61), Castor (EudraCT: 2014-000255-85), Tourmaline MM-1 (EudraCT: 2011-005496-17), and Admyre (EudraCT: 2009-016138-29).

In a second analysis, all RRMM patients were reviewed virtually for the whole 3-year time frame, utilizing trials that were actively recruiting internationally and that later resulted in drug approval. Every single relapse was then analyzed for all of the I/E criteria in the protocol. In addition to the above-mentioned Castor, Relapse, and Tourmaline MM-1 trials, the Pollux trial (EudraCT: 2013-005525-23) and the Eloquent-2 trial (EudraCT: 2010-020347-12) were analyzed.

Patient records were reviewed for the date of diagnosis and the date of relapse and refractory disease with their concurring lab results (whole blood count, kidney and liver function, and heavy and light chains). Additionally, myeloma classification according to IMWG criteria, drugs received, and trial participation or reason for exclusion were recorded. Concomitant disease and medical history were analyzed, including neuropathy, hepatitis, HIV, cardiac disease, other neoplasms, allergies, and GVHD. ECOG Performance Status ≤2 and the ability to consent to trial and/or the adherence to trial schedule, to monitor birth control, pregnancy, or nursing were not analyzed because they were either not documented, or because it was impossible to retrace these criteria retrospectively.

All trial protocols were published under www.clinicaltrials.gov (accessed on 8 March 2022). Table S1 gives a summary of all protocols reviewed.

All of the data were added to a spreadsheet and sorted for the desired criteria. For every patient, we documented relapsed/refractory disease, exposure to the study drug(s), time point of relapsed/refractory disease, and exposure. Totals, medians, and charts were calculated using Microsoft Excel.

When we documented therapy lines and relapses, all events were counted. This included patients who relapsed more than once within a year or patients with previous therapies who were sent to our center for evaluation. Thus, patients could be counted more than once, so that the total of therapy lines and relapses exceeded our cohort of 411 patients (Figure S2).

## 3. Results

### 3.1. Patient Characteristics

The initial cohort of patients treated at our institution in the years 2014, 2015, and 2016 contained 634 patients in our database with a diagnosis of plasma cell dyscrasia. However, 223 patients had to be excluded from analysis: 79 patients were diagnosed with monoclonal gammopathy of unknown significance (MGUS), 19 patients with smoldering myeloma, and 20 patients with AL-amyloidosis. Forty-nine patients came in for a second opinion only or were lost to follow-up. The data of 56 patients were incomplete (Figure S2).

The analyzed cohort contained 411 patients: 250 male (61%) and 161 female (39%), with a median age of 60 years (an age range of 25–87). A total of 291 patients (71%) were younger than 65 years.

In total, 178 patients (43%) were classified as stage I according to the ISS classification, 110 patients (27%) as stage II, and 104 patients (25%) as stage III (data were missing for 19 patients). FISH analysis was available for 167 patients: 132 patients showed standard risk and 35 patients showed high-risk genetics (according to IMWG criteria, including Del17p, t (4;14), t (14;16), as determined by FISH [16]). Thus, 58 patients (35%) could be classified as stage I according to the R-ISS classification, 63 (38%) as stage II, and 46 (28%) as stage III.

A total of 311 patients (76%) received high-dose melphalan with autologous stem cell transplant. A total of 180 patients (44%) received radiation therapy.

### 3.2. Therapy Lines and Progressions from 2014 to 2016

During our assigned 3-year time frame, 643 patients were treated with first-line therapy, 258 patients with second-line therapy, 60 patients with third-line therapy, 40 patients with fourth-line therapy, 25 patients with fifth-line therapy, 20 patients with sixth-line therapy, 12 patients with seventh-line therapy, 5 patients with eighth-line therapy, and 2 patients during each of the therapy lines 9–12 (Figure S3a).

Over the same time frame, 107 patients showed progressive disease during first-line therapy, 57 patients during second-line therapy, 45 patients during third-line therapy, 28 patients during fourth-line therapy, 16 patients during fifth-line therapy, 14 patients during sixth-line therapy, 7 patients during seventh-line therapy, 3 patients during eighth-line therapy, and 2 patients during each of therapy lines 9–11 (Figure S3b).

Overall, we treated 378 patients in 2014, 418 in 2015, and 428 in 2016. We recorded 87 relapses in 2014, 93 in 2015, and 103 in 2016. There were 60 newly diagnosed patients in 2014, 65 in 2015, and 38 in 2016.

The median number of prior therapy lines was two lines. The median time to progression for the first-line therapy was 1035 days. The median time to progression for the second-line therapy was 593 days. The median time to progression for the third-line therapy was 380 days, and for fourth-line therapy, 259 days.

Since patients could relapse or change therapy lines more than once a year, these numbers exceeded our cohort of 411 patients.

### 3.3. Study Participation—Real-World Data

A total of 411 patients were analyzed for the possibility of inclusion within one of the actively recruiting trials at the University Hospital Duesseldorf. Overall, 19% were eligible and enrolled to trial, 58% were not eligible, and 23% would have been eligible according to I/E criteria but declined to consent and were not screened.

When we grouped the patients who did not consent according to their age, we found 12% to be in the 40–50-year group, 29% of patients in the 50–60 group, and 59% of patients in the 60–70 group.

### 3.4. First-Line Trials

The GMMG-HD6 and LenaMain trials were able to recruit 38% and 46% of the cohort, respectively. A total of 51% (HD6) and 43% (LenaMain) did not fit the I/E criteria. In total, 11% (HD6) and 11% (LenaMain) of patients could have been included but did not consent to participation.

**Figure 1:**
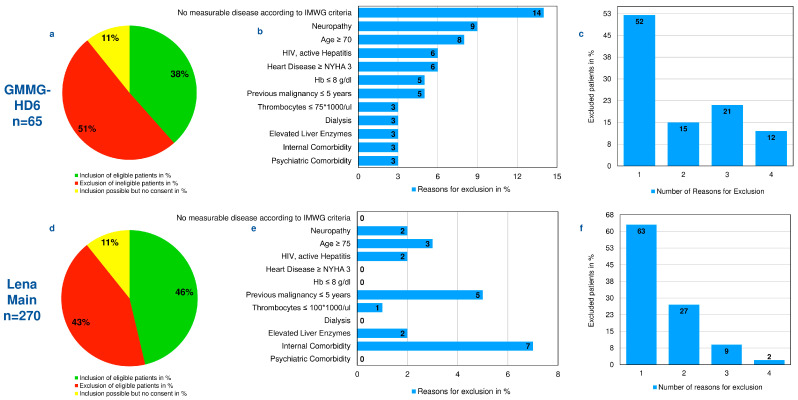
First-line trials, HD6 and LenaMain. (**a**,**d**) Patients included, excluded, and inclusion possible but no consent given by the patient. (**b**,**e**) Reasons for exclusion, in %. (**c**,**f**) Number of reasons for exclusion, in %.

The majority of reasons for non-eligibility were patient-associated: 14% did not meet the IMWG criteria for measurable disease according to IMWG criteria. Other reasons included pre-existing polyneuropathy ≥ II°(2–9%), age ≥ 70 years (3–8%), active infection with HIV or hepatitis virus (2–6%), or heart disease ≥ NYHA III° (6%). Of those non-eligible, 5% suffered from a previous malignancy within the last 5 years. The majority of patients (52% and 63%) displayed only one reason for exclusion (Figure 1a–f).

### 3.5. Second-Line Trials

**Figure 2:**
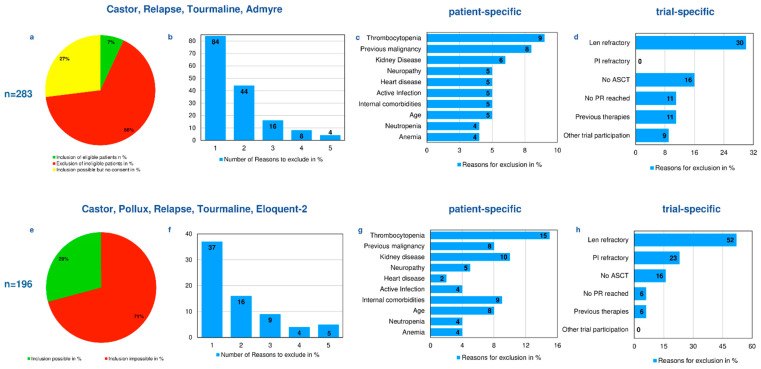
Second-line trials. (**a**) Patients included, excluded, and inclusion possible. (**b**) Number of reasons why patients had to be excluded. (**c**) Patient-specific reasons for exclusion. (**d**) Trial-specific reasons for exclusion. (**e**) Virtual view for if patients could have hypothetically been included in internationally recruiting trials. (**f**) Number of reasons for exclusion. (**g**) Patient-specific reasons for exclusion. (**h**) Trial-specific reasons for exclusion.

The second-line trials, Castor, Relapse, Tourmaline, and Admyre, were able to recruit 7% of the cohort, while 66% did not fit the I/E-criteria. A total of 27% of patients could have been recruited according to I/E criteria, but they did not consent to participation. The majority of reasons for non-eligibility were trial-related, with 30% being refractory to the trial medication, or 11% not having reached at least a partial remission to prior therapy. Patient-related factors included low platelet counts (9%), a previous malignancy (8%), or renal failure (6%). The vast majority of patients displayed only one reason for exclusion (84%) (Figure 2a–d).

### 3.6. Study Participation—Virtual Analysis

Of the original cohort of 411 patients, 196 were identified that had suffered between one to three relapses during the assigned time frame. Our virtual view on the second-line trials, Castor, Pollux, Relapse, Tourmaline, and Eloquent-2, showed that these trials would have been able to recruit 29% of our cohort within a 3-year time frame, while 71% did not fit the I/E-criteria. The majority of reasons for non-eligibility were trial-related, with 75% being refractory to the trial medication, 16% not having received a transplant, and 6% not having reached at least a partial remission to prior therapy. Patient-related factors included low platelet counts (15%), a previous neoplastic disease (8%), or renal failure (10%). The majority of patients (37% and 16%) displayed only one or two reasons for exclusion (Figure 2e–h).

## 4. Discussion

Conducting randomized clinical trials (RCTs) is fundamental to the advancement of new cancer treatments that can have major impacts on patient outcomes. However, the vast majority of adult cancer patients do not participate in trials, with participation rates ranging from 2% to (more recently) 8% [17,18]. Barriers to trial participation have been extensively studied, but the rate of trial participation has not changed substantially over time. There is, however, evidence that trial participation correlates with improved survival and reduced mortality [15,19,20,21].

Based on a cohort of 411 myeloma patients treated at our center, we were able to show that 53% of NDMM patients were eligible for a first-line trial, and of these, 80% were included. However, only 34% of RRMM patients were eligible for a trial, and of these, only 22% were included. A virtual examination of second-line trials recruiting worldwide during a 3-year time frame confirmed eligibility in only 29% of our cohort.

Thus, our center recruited more MM patients than the average oncology trial. Unger et al. have shown that trial participation rates are usually higher in academic centers than in community centers [17]. Additionally, our patient cohort was somewhat biased, since the patients that are referred to our center are usually younger and fitter than the standard MM population. Older patients are often excluded from trials to ensure safety and to avoid comorbidities meddling with results [22,23]. However, several chemotherapy-containing trials have shown no indications of increased toxicity in elderly patients when they were appropriately selected [24,25]. Geriatric assessment and prediction models that are capable of estimating risk—although time-consuming and tedious—might help physicians to better evaluate the fitness levels of elderly patients [26]. Mobility also seems to be a major limiting factor for elderly patients. Kessel et al. have shown that 25% of patients refuse consent because of the distance to the treatment center [27]. Especially frail patients or patients with bone disease tend to be more reluctant due to logistical barriers, because RCTs require more visits and therefore more frequent travel sessions. We found that twice as many patients between 60 and 70 years of age did not consent to trial participation compared to patients aged between 50 and 60 years of age. Lewis JH et al. suggested that that the participation of older patients in clinical trials would approach 60% if protocol exclusions related to functional status were relaxed [28]. This approach could be even further strengthened by installing more satellite centers or by appointing study nurses to travel to patient consultations. Additionally, virtual visits could alleviate the need to travel long distances [29].

Eligibility criteria are a crucial instrument for selecting a homogeneous patient population that will maximize the ability to identify possible treatment effects without dilution by factors that are unrelated to treatment [13]. However, strict RCT criteria often deselect patients, leading to limited recruitment and a cohort that is not representative of a real-world patient population [14].

Our work shows that reasons that render a patient ineligible are usually a combination of both trial-related and patient-related factors. First-line trials are generally designed to include most newly diagnosed MM patients. Thus, we have found that more patient-related factors for ineligibility exist in first-line trials, since newly diagnosed MM patients often present with advanced stages of the disease and more disease-related comorbidities. Relapsed/refractory patients, in contrast, are often affected by trial-related exclusion criteria, since these RCTs often contain higher safety standards, plus previously received therapies or refractoriness to the trial medication as exclusion criteria.

We found that most patient-specific exclusion criteria included low platelet counts, renal disease, or previous neoplasia. With data from the MM Connect registry, Shah et al. found that lower platelet levels and creatinine levels > 2.5 mg/dL, as well as previous incidence of malignancy, were associated with a greater risk of serious adverse events (SAE) within RCTs [8]. However, this analysis stemmed from a database that collected SAEs across all lines of therapy. Chari et al. found only a platelet count < 75 K/L to be a significantly adverse prognostic indicator [30]. Dimopoulos et al. showed that the introduction of novel agents significantly improved the survival of patients with severe renal impairment, suggesting that more patients with reduced renal function could be recruited to clinical trials [31]. Notably, patients with previous neoplasia should be considered for trials when the risk of the malignancy or its treatment in interfering with safety or efficacy endpoints of the trial appears manageable [13]. Therefore, patients with lower platelet counts, mild renal disease, or previous neoplasia could particularly benefit from trial participation. Modifying these specific trial criteria would lead to a population that is more representative of the real-world MM population and would accelerate enrolment without affecting the integrity of the trial or compromising patient safety.

Trial-specific exclusion criteria are more difficult to challenge, as they are required for patient safety. However, recruitment could be improved laterally by improving networking between myeloma working groups, and by cancer centers and oncology practices providing more information in greater detail on current trials to reach more eligible patients. Increasingly, social media platforms are being used as a new tool for researchers and potential participants to communicate about clinical trials [32].

MM is a heterogeneous disease with a heterogeneous patient population [33]. Therefore, individual patients may view the concept of ‘efficacy’ quite differently [34]. Younger patients might prioritize life expectancy and may opt for a more intensive treatment to elicit a deeper response by enrolling in an RCT with novel agents [35]. In contrast, elderly/frail patients may prefer disease control and maintaining their quality of life (QoL), as well as treatment convenience and the ability to continue with daily activities [36,37,38,39]. Older patients with more advanced disease stages tend to trade off efficacy for tolerability [40]. This is reinforced by our data, which showed that twice as many people over 60 years of age decided against a trial participation compared to those under 60 years old.

Patient groups may not be the only categories that consider treatment preferences and the weighting of factors differently. Patients often change their priorities at different stages over the course of their treatment [41]. To the best of our knowledge, our data have shown for the first time that patients tend to become more reluctant to participate in trials over the course of the disease. A total of 11% of newly diagnosed patients in our cohort did not consent to trial, while 28% did not consent in the relapsed/refractory setting. Unger et al. found that the most common reason for patients not enrolling was the wish to control their treatment choice [17]. Relapsed patients may often fear the exacerbation of previous side effects by new treatments. Unger et al. also found that a small number of patients did indeed dislike participation in an experiment, including the dislike of having treatment being determined by random assignment [17]. This is a common fear and could be alleviated by designing control arms from which patients can still benefit significantly. Many studies have also provided crossovers or 2:1 randomization to address this issue.

Overall, many reservations can be addressed by having physicians provide the best possible information and allow for open discussion, with a focus on the patient’s needs and fears. However, clinical trials require large amounts of resources from institutions, with a heavy commitment to consuming various resources. A much greater impact may be achieved by addressing the numerous obstacles that occur prior to patient–physician interaction. One example would be the provision of better support for travel to the treatment center. Also extended negotiations between institutions regarding compensation have been found to curtail recruitment time and have a harmful effect on trials, compared to any savings in financial expenditure. Thus, trial design improvement is imperative for the provision of better care to patients and the advancement of new treatments.

One limitation of our study was the use of a fairly homogenous cohort that comprised mostly White Caucasians. This was not a fair representation of the general MM patient cohort. The incidence of MM has increased in recent years, with a higher incidence in Non-Hispanic Black (NHB) patients [42,43,44,45]. Patients with a high degree of African ancestry are more likely than those with a high degree of European ancestry to have translocations involving the immunoglobulin heavy-chain gene on chromosome 14, which suggests a fundamental difference in disease biology between NHB and Whites [46]. However, it is known that NHB patients are underrepresented in clinical trials for MM [47]. Amongst trials including novel drugs, a mere 4.5% were NHB, and this subgroup comprised only 1.8% of the study population in international trials [48,49]. These reasons are likely multifactorial, but they could include health literacy, mistrust in the medical system, a lack of access to participating centers, cultural or religious beliefs, and a fear of side effects. Expanding the inclusion criteria could be one strategy for increasing diversity in clinical trials. Support by bilingual personnel could also be useful. More importantly, community outreach and education is needed to overcome these barriers in order to provide better care with modern therapies and improve patient survival, especially for minorities.

## 5. Conclusions

Despite a higher-than-average recruitment rate, this analysis shows that only a subset of the multiple myeloma patient population treated at our center had access to clinical trials. Strict inclusion/exclusion criteria rendered a majority of patients ineligible due to study-specific criteria such as prior therapies and refractory disease. Kidney disease, blood counts, and previous malignancy represented the most common patient-specific criteria.

Relapsed/refractory patients are much less inclined to participate in a clinical trial than newly diagnosed myeloma patients due to shifted expectations and priorities.

It is imperative that inclusion/exclusion criteria are adjusted accordingly, and that access to clinical trial information and support are increased both before and during the trials, in order to improve access to novel agents for a broader patient population.

## Data Availability

All trial protocols analyzed were published under www.clinicaltrials.gov (accessed on 8 March 2022).

## Supplementary Materials

The following supporting information can be downloaded at: https://www.mdpi.com/article/10.3390/cancers14092147/s1. Table S1: Clinical trial characteristics and summary; Figure S1: Timeline: (a) analysis based on real-world data from a cohort of NDMM and RRMM patients; Figure S2: Patient characteristics: (a) cohort characteristics: 223 patients were excluded from analysis for reasons mentioned; Figure S3: Patients and relapses per therapy line: lines of treatment, 2014–2016: (a) total number of patients per therapy line (TL); (b) number of progressions per therapy line.
