# Supplementary material for: Opportunities for Participation in Randomized Controlled Trials for Patients with Multiple Myeloma: Trial Access Depends on Restrictive Eligibility Criteria and Patient Expectations
Source: Cancers (Basel). 2022 Apr 26;14(9):2147. doi: 10.3390/cancers14092147 (PMC9106039; doi:10.3390/cancers14092147)
Supplement: Supplementary file 1 [file cancers-14-02147-s001.zip › cancers-1638809-supplementary.pdf]

cancers

Table S1. Clinical trial characteristics and summary.

Trial Number	Trial Name	Summary	Reference	Major inclusion/exclusion criteria
**EudraCT: 2011-005496-17; NCT: NCT01564537**	Tourmaline MM-1	A Phase 3, Randomized, Double-Blind, Multicenter Study Comparing Oral MLN9708 Plus Lenalidomide and Dexamethasone Versus Placebo Plus Lenalidomide and Dexamethasone in Adult Patients With Relapsed and/or Refractory Multiple Myeloma	Moreau et al. N Engl J Med 2016; 374:1621-34	MM with 1-3. relapse<br>Not refractory to Lenalidomide or proteasome inhibitor<br>refractory toward the last therapy line, relapse during last therapy<br>Serum IgG M-Protein ≥1 g/dL, Urine M-Protein ≥200 mg/24h, Serum FLC ≥10 mg/ dL und and abnormal I/U ratio<br>ANC ≥1.0x10^9/L, Thrombocytes ≥75x10^9/L, Hb ≥8 g/dl, Kreatinin-Clearance ≥30 ml/min, Bilirubin ≤1.5 xULN, AST und ALT ≤3 xULN<br>ECOG ≤2<br>Previous malignancy ≥ 2 years except basal and squamous cell skin cancers or carcinoma in situ
**EudraCT: 2009-016138-29; NCT: NCT01102426**	Admyre	Randomized, Multicenter, Open-label, Phase III Study of Plitidepsin in Combination with Dexamethasone vs. Dexamethasone Alone in Patients with Relapsed/Refractory Multiple Myeloma	Spicka et al. Ann Hematol. 2019; 98:2139-2150	3-6 relapses or refractory disease<br>No previous therapy with Bortezomib or Lenalidomide<br>Urine M-Protein ≥200 mg/24h or Serum FLC ≥10 mg/ dL<br>ANC ≥1.0x10^9/L, Thrombocytes ≥50x10^9/L, Hb ≥8.5 g/dl, Kreatinin-Clearance ≥30 ml/min, Bilirubin ≤1 xULN, AST und ALT ≤3.5 xULN, Serumcalcium ≥11.5 mg/dl<br>ECOG ≤2<br>PNP ≥ II°
**EudraCT: 2013-005525-23; NCT: NCT02076009**	Pollux	Phase 3 Study Comparing Daratumumab, Lenalidomide, and Dexamethasone (DRd) vs Lenalidomide and Dexamethasone (Rd) in Subjects With Relapsed or Refractory Multiple Myeloma	Dimopoulos et al. N Engl J Med 2016; 375:1319-1331	at least one previous therapy line with PR or better<br>progression according to IMWG-criteria<br>no previous therapy with Daratumumab or other CD38 antibody<br>not Lenalidomide refractory<br>ECOG ≤2<br>PNP ≥ II°<br>Serum M-Protein ≥1,0 g/dL or Urine M-Protein ≥200 mg/24h or IgA ≥0,5 g/dL or Urine M-Protein ≥200 mg/24h or Serum FLC ≥10 mg/ dL und, abnormal I/U ratio<br>ANC ≥1.0x10^9/L, Thrombocytes ≥75x10^9/L Hb ≥7,5 g/dl, Kreatinin-Clearance ≥30 ml/min, Bilirubin ≥2 xULN, AST und ALT ≥2,5 xULN, corrected Serumcalcium ≥14 mg/dl, ≥3,5 mmol/L<br>previous malignancy ≥ 3 years except carcinoma in situ of breast or cervix, basal and squamous cell skin cancers, non-invasive tumors
**EudraCT: 2010-020347-12; NCT: NCT01239797**	Eloquent-2	A Phase 3, Randomized, Open Label Trial of Lenalidomide/dexamethasone With or Without Elotuzumab in Relapsed or Refractory Multiple Myeloma	Lonial et al. N Engl J Med 2015; 373:621-631	MM with 1-3. relapse<br>Not Lenalidomide refractory<br>Previous therapy with Lenalidomide if best response ≥ PR, not refractory during Len treatment or within 9 months after discontinuation, progression ≥ 9 months after Len discontinuation<br>Serum IgG M-Protein ≥0.5 g/dL, Serum IgA M-Protein ≥0.5 g/dL, Serum IgM M-Protein ≥0.5 g/dL, Serum IgD M-Protein ≥0.05 g/dL, Urine M-Protein ≥200 mg/24h<br>ANC ≥1.0x10^9/L, Thrombocytes ≥75x10^9/L, Hb ≥8 g/dl, Kreatinin-Clearance ≥30 ml/min, Bilirubin ≥2 xULN, AST/ALT ≥3 xULN, Serumcalcium ≥11.5 mg/dl<br>ECOG ≤2<br>Previous malignancy ≥ 5 years except basal and squamous cell skin cancers
**Trial Number**	**Trial Name**	**Summary**	**Reference**	**Major inclusion/exclusion criteria**
**EudraCT: 2007-003945-33; NCT: NCT00891384**	LenaMain	A randomised comparison of daily 25 mg versus 5 mg lenalidomide as maintenance therapy after high-dose therapy and autologous stem cell transplantation in patients with multiple myeloma	Fenk et al. Clin Cancer Res 2020; 26: 5879-5886	Autologous stem cell transplant within 90-120 days<br>ANC ≥1.0x10^9/L, Thrombocytes ≥100x10^9/L, Bilirubin ≤2,5 mg/dl, AST and ALT ≤3x ULN<br>ECOG ≤2<br>Patients with kidney disease receive adjusted dose<br>previous malignancy ≥ 5 years except carcinoma in situ of breast or cervix, basal and squamous cell skin cancers
**EudraCT: 2014-003079-40; NCT: NCT02495922**	GMMG-HD6	A randomized phase III trial on the effect of elotuzumab in VRD induction / consolidation and lenalidomide maintenance in patients with newly diagnosed myeloma	Salwender et al. BMC Cancer 2019; 19:504.	Serum M-Protein ≥10 g/l (IgA ≥0,5 g/l), Urine Bence-Jones-Protein ≥200 mg/24h, FLC Level ≥10 mg/dL if sFLC is abnormal<br>ANC ≥1.0x10^9/L, Thrombozyten: ≥75x10^9/L, Bilirubin: ≤1,8 mg/dl, AST und ALT ≤2,5 ULN, Hb ≥8 g/dl<br>ECOG ≤3<br>previous malignancy ≥ 5 years except carcinoma in situ of breast or cervix, basal and squamous cell skin cancers<br>no dialysis
**EudraCT: 2009-013856-61; ISRCTN16345835**	Relapse	A phase III national, multicentre, randomized open-label study with Lenalidomide/Dexamethasone versus Lenalidomide/Dexamethasone and autologous stem cell transplantation followed by Lenalidomide maintenance for patients with relapsed Multiple Myeloma	Goldschmidt et al. Leukemia 2021; 35:1134-1144.	MM with 1-3. relapse<br>If previous treatment with Lenalidomide: no remission (stable disease) or PD during or ≤60 days after Len discontinuation, if response ≥MR: PD ≤6 months after Len<br>ECOG ≤2<br>ANC ≥1.0x10^9/L, Thrombocytes ≥75x10^9/L, Kreatinin-Clearance ≥30 ml/min, Bilirubin ≤2 xULN, ALT ≤3 xULN<br>previous malignancy ≥ 5 years except carcinoma in situ of breast or cervix, basal and squamous cell skin cancers
**EudraCT: 2014-000255-85 NCT: NCT02136134**	Castor	Phase 3 Study Comparing Daratumumab, Bortezomib and Dexamethasone (DVd) vs Bortezomib and Dexamethasone (Vd) in Subjects With Relapsed or Refractory Multiple Myeloma	Palumbo et al. N Engl J Med 2016; 375:754-766	MM with 1-3. relapse<br>previous therapy with Daratumumab or other CD38 antibody<br>Serum IgG ≥1,0 g/ dL or Serum IgA, IgD, IgE, IgM ≥0,5 g/dL or Urine M-Protein ≥200 mg/24h or Serum FLC ≥10 mg/dL and abnormal I/U ratio<br>ECOG ≤2<br>PNP ≥ II°<br>ANC ≥1.0x10^9/L, Thrombocytes ≥75x10^9/L, Hb ≥7,5 g/dl (no Transfusion $<$72h before trial start), Kreatinin-Clearance ≥20 ml/min, Bilirubin ≤1,0 mg/dl, AST und ALT ≥2,5 xULN, corrected Serumcalcium ≥14 mg/dl / ≥3.5 mmol/L<br>previous malignancy ≥ 3 years except carcinoma in situ of breast or cervix, basal and squamous cell skin cancers, non-invasive tumors

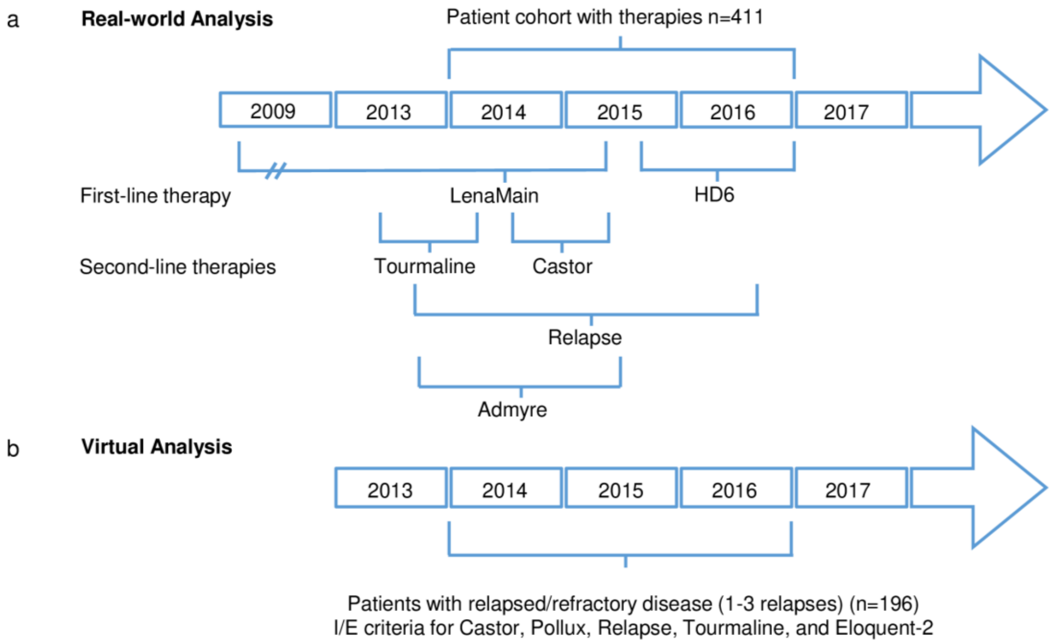

Figure S1. Time line: a) Analysis based on real-world data from a cohort of NDMM and RRMM patients. First-line trials LenaMain and GMMG-HD6, and second-line trials Relapse, Castor, Tourmaline MM-1 and Admyre. b) In a second analysis, all RRMM patients were reviewed virtually during a 3 year time frame. In addition to Castor, Relapse, and Tourmaline MM-1 this entailed the Pollux and the Eloquent-2 trial.

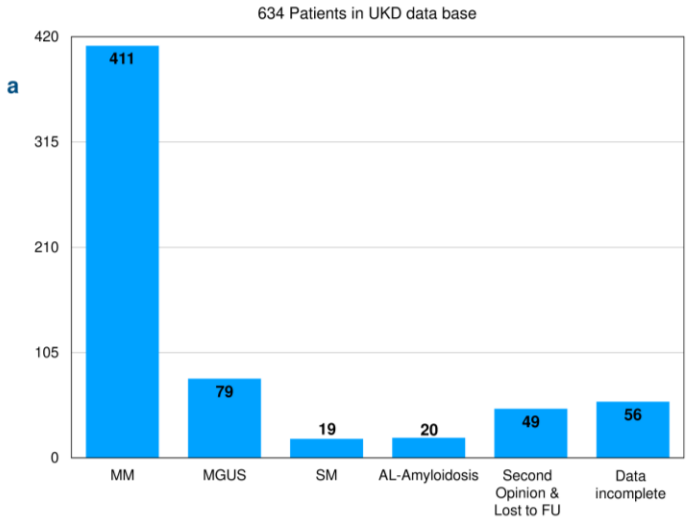

b

Patient Characteristics n=411

Gender n (%)	male : female	250:161 (61:39)
Age (years)	median (range)	58.9 (25-87)
Subtype n (%)	IgG	244 (60)
	IgA	74 (18)
	IgM	3 (1)
	Light chain	85 (21)
	Other	5 (2)
Creatinine n (%)	< 2 mg/dl	304 (74)
	> 2 mg/dl	52 (13)
ISS Stage n (%)	I	178 (43)
	II	110 (27)
	III	104 (25)

Figure S2. Patient characteristics: a) cohort characteristics: 223 patients were excluded from analysis for reasons mentioned. Multiple Myeloma (MM), Monoclonal gammopathy of unknown significance (MGUS), Smoldering Myeloma (SM), FU=Followup b) patient characteristics.

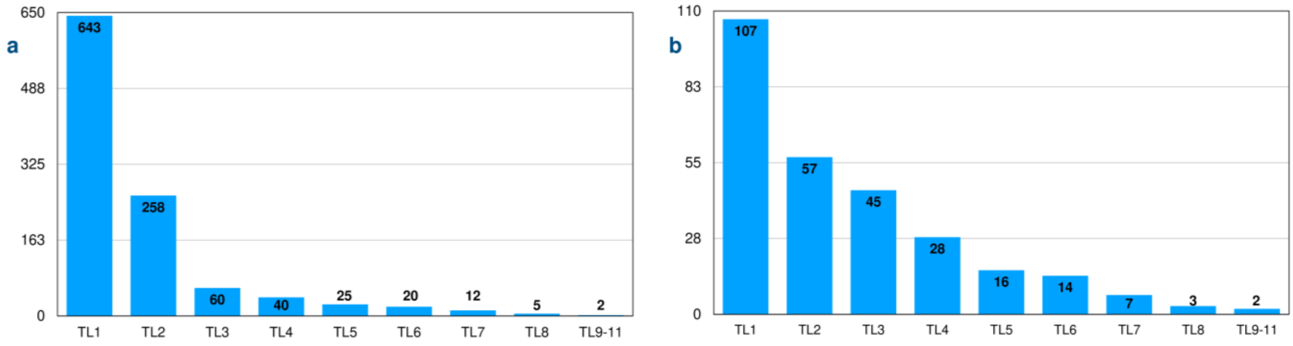

Figure S3. Patients and Relapses per Therapy Line: Lines of treatment 2014-2016 a) Total number of patients per therapy line (TL) b) Number of progressions per therapy line. Since patients shift between therapy lines due to progressive disease, they can be counted more than once.
